# Oxidative and Molecular Responses in *Capsicum annuum* L. after Hydrogen Peroxide, Salicylic Acid and Chitosan Foliar Applications

**DOI:** 10.3390/ijms140510178

**Published:** 2013-05-15

**Authors:** Laura Mejía-Teniente, Flor de Dalia Durán-Flores, Angela María Chapa-Oliver, Irineo Torres-Pacheco, Andrés Cruz-Hernández, Mario M. González-Chavira, Rosalía V. Ocampo-Velázquez, Ramón G. Guevara-González

**Affiliations:** 1 Biosystems Engineering Group, School of Engineering, Queretaro Autonomous University, C.U Cerro de las Campanas, S/N, colonia Las Campanas, CP 76010, Santiago de Querétaro, Querétaro, Mexico; E-Mails: lauralyo@yahoo.com.mx (L.M.-T.); dalia.azul@gmail.com (F.D.D.-F.); angellox2040@hotmail.com (A.M.C.-O.); irineo.torres@uaq.mx (I.T.-P.); andrex1998@hotmail.com (A.C.-H.); rosov05@yahoo.com.mx (R.V.O.-V.); 2 Biotechnology Group, National Institute for Forestry, Agriculture and Livestock Research (INIFAP), Celaya-San Miguel de Allende, km 6, CP 38010, Celaya, Guanajuato, Mexico; E-Mail: gonzalez.mario@inifap.gob.mx

**Keywords:** reactive oxygen species, oxidative stress, elicitors, oxidative stress dynamic, hydrogen peroxide, salicylic acid, chitosan, catalase, phenylalanine ammonia lyase

## Abstract

Hydrogen peroxide (H_2_O_2_) is an important ROS molecule (Reactive oxygen species) that serves as a signal of oxidative stress and activation of signaling cascades as a result of the early response of the plant to biotic stress. This response can also be generated with the application of elicitors, stable molecules that induce the activation of transduction cascades and hormonal pathways, which trigger induced resistance to environmental stress. In this work, we evaluated the endogenous H_2_O_2_ production caused by salicylic acid (SA), chitosan (QN), and H_2_O_2_ elicitors in *Capsicum annuum* L. Hydrogen peroxide production after elicitation, catalase (CAT) and phenylalanine ammonia lyase (PAL) activities, as well as gene expression analysis of *cat1*, *pal*, and pathogenesis-related protein 1 (*pr1*) were determined. Our results displayed that 6.7 and 10 mM SA concentrations, and, 14 and 18 mM H_2_O_2_ concentrations, induced an endogenous H_2_O_2_ and gene expression. QN treatments induced the same responses in lesser proportion than the other two elicitors. Endogenous H_2_O_2_ production monitored during several days, showed results that could be an indicator for determining application opportunity uses in agriculture for maintaining plant alert systems against a stress.

## 1. Introduction

Plants are frequently exposed to different environmental stresses, which can be both biotic and/or abiotic. These stresses cause biochemical alterations as generation of hydrogen peroxide (H_2_O_2_) resulting in an early response of the plant defense mechanism [1–4]. The oxidative burst, the generation of reactive oxygen species (ROS) in response to microbial pathogen attack, is a ubiquitous early part of the resistance mechanisms of plant cells. H_2_O_2_ is a form of Reactive Oxygen Species (ROS) which are generated as a result of oxidative stress, and it is involved in the control and regulation of biological processes, such as growth, cell cycle, programmed cell death, hormone signaling, biotic/abiotic stress responses, and development [2,4–7]. The aforementioned research suggests that during the course of evolution, plants were able to achieve a high control degree over ROS toxicity, through a highly balanced and tightly coordinated network of at least 152 genes which encode both ROS-producing and ROS-scavenging enzymes [6]. For this reason, ROS molecules have been used as signaling molecules and accordingly, the interplay between the ROS-producing and ROS-scavenging pathways will determine the intensity, duration and localization of the ROS signals [6]. Usually, high intensity cellular signaling via ROS is generated by biotic stress, particularly in plant-pathogen interactions. However, this signaling cascade can also be activated by the use of elicitors, stable molecules that induce an immune defense response in plants, similar to that generated by microorganism-associated molecular patterns (MAMPs) [8–10]. Elicitor-induced plant signaling, serves as a guide to a series of intracellular events that end in the activation of transduction cascades and hormonal pathways, which trigger induced resistance and consequently activate plant immunity to environmental stresses [9–11]. Many substances have been discovered that work as elicitors [12]. Some examples are jasmonates, such as methyl jasmonate (MJ) and jasmonic acid (JA); other groups include salicylic acid (SA), benzothiadiazole (BTH), Etephon, hydrogen peroxide, and oligosaccharides such as chitosan, among other compounds [13]. Plant defensive mechanisms could be encouraged through the use of elicitors [14,15]. In fact, it is known that treatment of plants with elicitors, or attack with pathogens, causes a set of defense reactions such as the accumulation of defensive secondary metabolites in edible and inedible parts of plants, specific gene expression and enzymatic induction [13]. The effect of elicitors depends on many factors such as the concentration of the elicitor, time of elicitation, and stage in which elicitor is applied [16]. Also, elicitors can have a synergistic effect. Heredia and Cisneros-Zevallos [17] reported that a combination of ET and MJ on wounded lettuce, celery, red onions, carrots, and jicama tissues amplifies the stress response possibly because both stresses may share common signaling molecules. Thus, the aim of this work was to evaluate the effect of the SA, H_2_O_2_, and QN elicitors on H_2_O_2_ production, gene and enzymatic defense-related dynamic in *Capsicum annuum* L. Our results are discussed and concluded in terms of new possibilities of doses-scheme strategies in crop protection against biotic stresses resulting from the biochemical and molecular studies carried out in this research.

## 2. Results and Discussion

### 2.1. H_2_O_2_ Detection with DAB in *C. annuum* L. due to Elicitors’ Application

**Figure 1:**
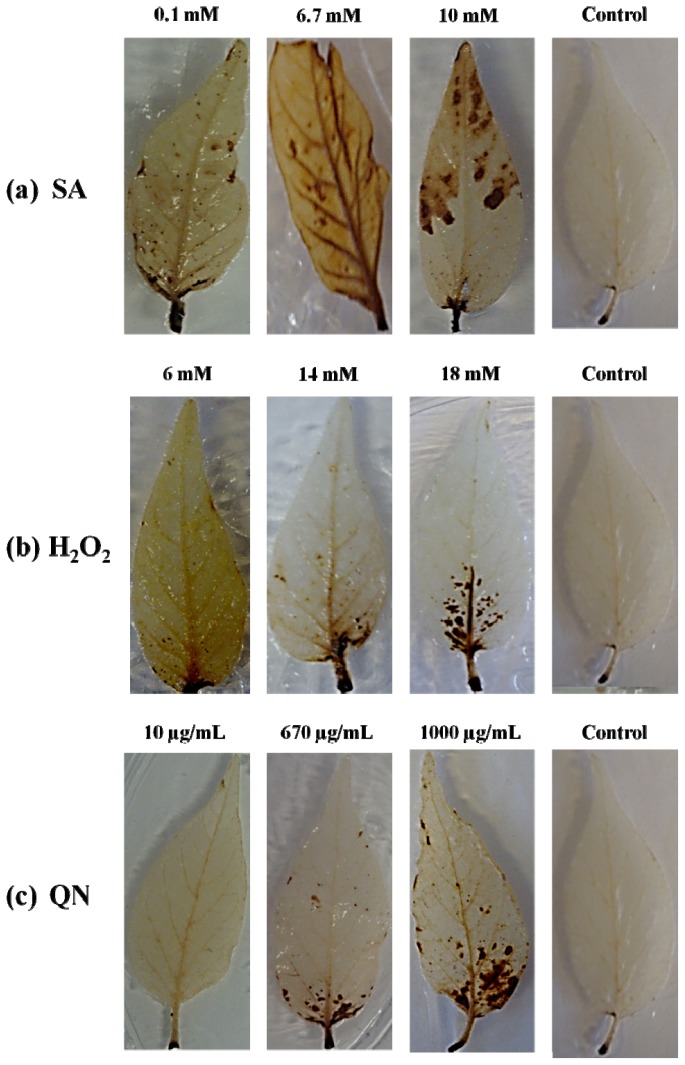
Hydrogen peroxide detection using DAB staining at 12 h post-application of elicitors in *C. annuum* L. var. Don Benito.

The H_2_O_2_ generation in leaves of *C. annuum* L. var. Don Benito due to the elicitors’ application was visually analyzed by staining with 3, 3′-Diaminobencidine (DAB) [18]. This staining polymerizes and turns deep brown in the presence of H_2_O_2_, and the intensity of the coloration and its localization can be visually assessed. H_2_O_2_ production in *C. annuum* L. leaves at 12 h post-application of SA (0.1, 6.7 and 10 mM), H_2_O_2_ (6, 14 and 18 mM) and QN (10, 670 and 1000 μg/mL) elicitors is shown in Figure 1. We observed a strong coloration induced with 6.7 and 10 mM SA concentration, in contrast with the 0.1 mM concentration where the signal was less intense. The same trend, of minor to major accumulation of hydrogen peroxide was observed, but in less intensity with applications H_2_O_2_ (14 and 18 mM) and QN (670 μg/mL and 1000 μg/mL) elicitors. The presence of endogenous hydrogen peroxide induced by foliar application of H_2_O_2_ (6 mM) elicitor is minimal compared with application of the other concentrations of this elicitor. Alternatively, it simply had no visible effect, at least with DAB staining, as was case of foliar application QN (10 μg/mL) elicitor. In general, the color was visibly observed at the base of the leaf, notably deeper in the tissue, and immediately appearing in primary and secondary veins from leaves (Figure 1). In contrast with the control, this was only sprayed with water, and where generation of H_2_O_2_ was not observed. Our results showed that exogenous application of SA, H_2_O_2_, and QN elicitors significantly induced hydrogen peroxide generation depending on the concentration of elicitor. This is an important point, because generally ROS molecules as hydrogen peroxide, and plant resistance to biotic stress are directly related to plant infection [19]. One example is reported by Guevara-Olvera *et al*. [20] where H_2_O_2_ production, evaluated by DAB staining, was significantly higher at 6 h post inoculation (hpi) with geminivirus in transgenic tobacco lines than expressing *Cch*GLP gene related to defense against biotic stress. Also, García-Neria *et al*. [21] showed a high accumulation of H_2_O_2_ at 6 hpi in PepGMV-inoculated leaves of resistant plants (*C. chinense* accession BG-3821) than in similar leaves from susceptible plants of *C. annuum* var. Sonora Anaheim. Our results also show the durability of the accumulation of H_2_O_2_ until 12 h post application elicitors. Thus, we can infer that the plant could be on alert for any kind of stress. This is likely because H_2_O_2_ is produced in response to a variety of stimuli, and mediates cross-talk between signaling pathways. Moreover, some elicitors may not require a receptor-based mechanism for their activity [1,22,23].

### 2.2. Quantitative Analysis of the H_2_O_2_ Endogenous Content in *Capsicum annuum* L

**Figure 2:**
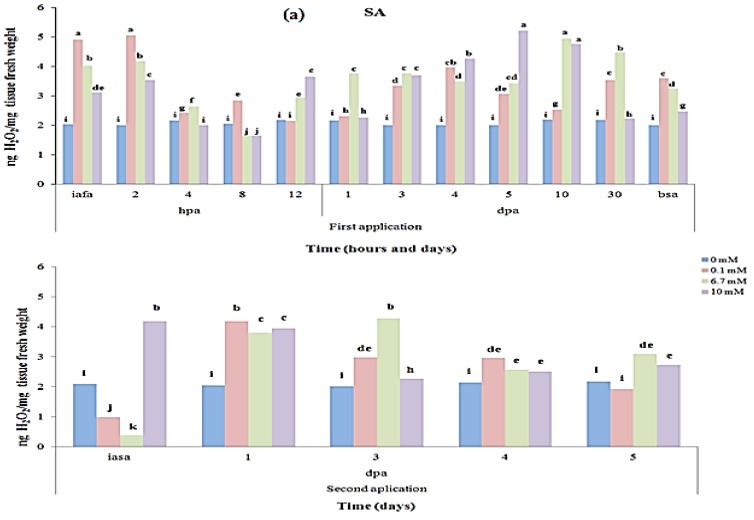

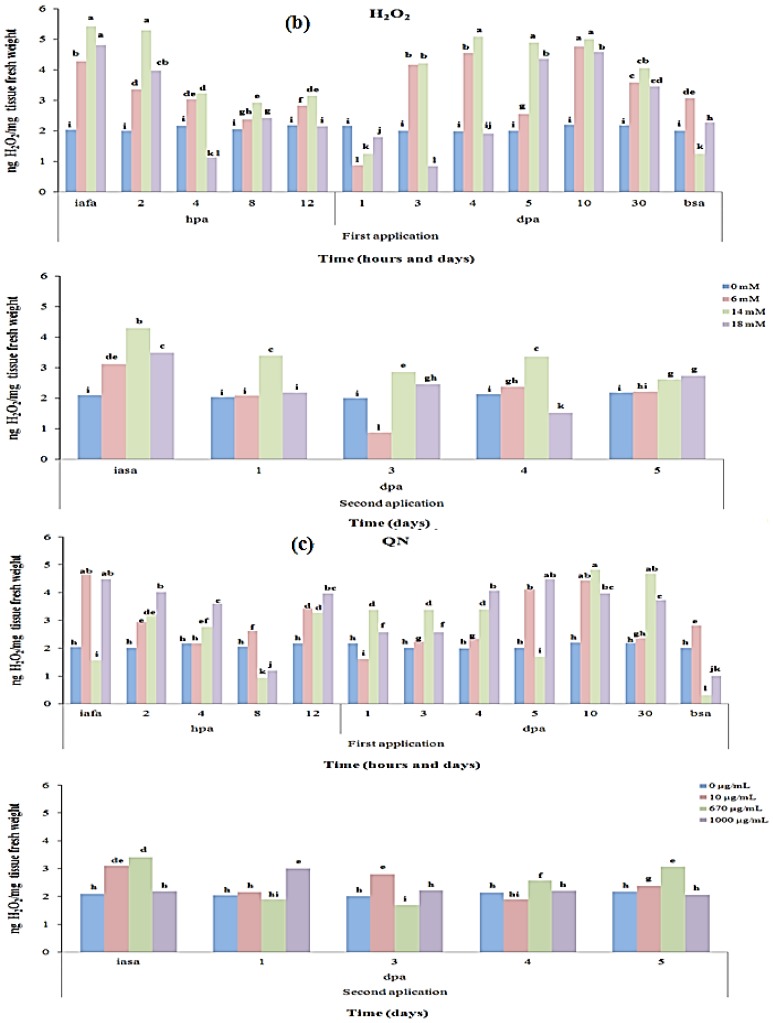
H_2_O_2_ production dynamic in *C. annuum* L. var. Don Benito as result of elicitors’ applications at two different times. H_2_O_2_ production generated by three concentrations of: (**a**) SA elicitor; (**b**) H_2_O_2_ elicitor; (**c**) QN elicitor. Elicitors’ application first at day zero, and second application at day 42 after first application of elicitors. Abreviations: iafa: immediately after first application; bsa: before second application; iasa: immediately after second application; hpa: hours post application; dpa: days post application.

Oxidative stress arises from an imbalance of metabolism and generation of ROS, and the extent of oxidative stress in a cell is determined by the amounts of superoxide, hydrogen peroxide, and hydroxyl radicals [24]. The amount of H_2_O_2_ generated by elicitor’s application in *C. annuum* L. was measured and a dynamic production of the molecule was shown over time, in order to establish the H_2_O_2_ production durability. H_2_O_2_ production dynamic by effect of SA (0.1, 6 and 10 mM), H_2_O_2_ (6, 14 and 18 mM) and QN (10, 670 and 1000 μg/mL) application is showed in Figure 2. It was shown that H_2_O_2_ production in *C. annuum* L. was significantly triggered in the early hours after elicitors’ application, compared to control. Moreover, in control treatments (0 mM elicitor), H_2_O_2_ levels were not significantly different during the evaluated periods. The monitoring of peroxide production was followed until day 30 after application of SA, H_2_O_2_ and QN elicitors, in order to estimate the H_2_O_2_ endogenous levels after the first elicitation. H_2_O_2_ production’s highest levels were localized from 4 to 30 days post-application (dpa) of elicitors’ application (Figure 2). It was also shown that H_2_O_2_ decreased until day 42 post first application (bsa) of elicitors (Figure 2). During day 42, we carried out a second application of elicitors in order to evaluate the H_2_O_2_ production levels when the plants are in a more developed stage. After second applications, H_2_O_2_ production level increased significantly, and differences were observed in H_2_O_2_ production among elicitors and their concentrations (Figure 2). Noteworthy that the half-life of exogenous 20 mM H_2_O_2_ is 2 min, and that after only 5 min, no H_2_O_2_ is detectable [25]. Therefore, the results shown refer only to endogen peroxide content caused by elicitors. H_2_O_2_ production was similar in SA (5.21 ng/mg tissue fresh weight, Figure 2a) and H_2_O_2_ (4.9 ng/mg tissue fresh weight, Figure 2b). In QN applications, H_2_O_2_ production reached 4.5 ng/mg fresh weight, which coincides with Lin *et al*. [26] who showed an inhibition of the chitosan-mediated increase in the H_2_O_2_ levels that led to a lower expression of glucanase and chitinase transcripts in rice.

### 2.3. CAT and PAL Enzyme Activity

**Figure 3:**
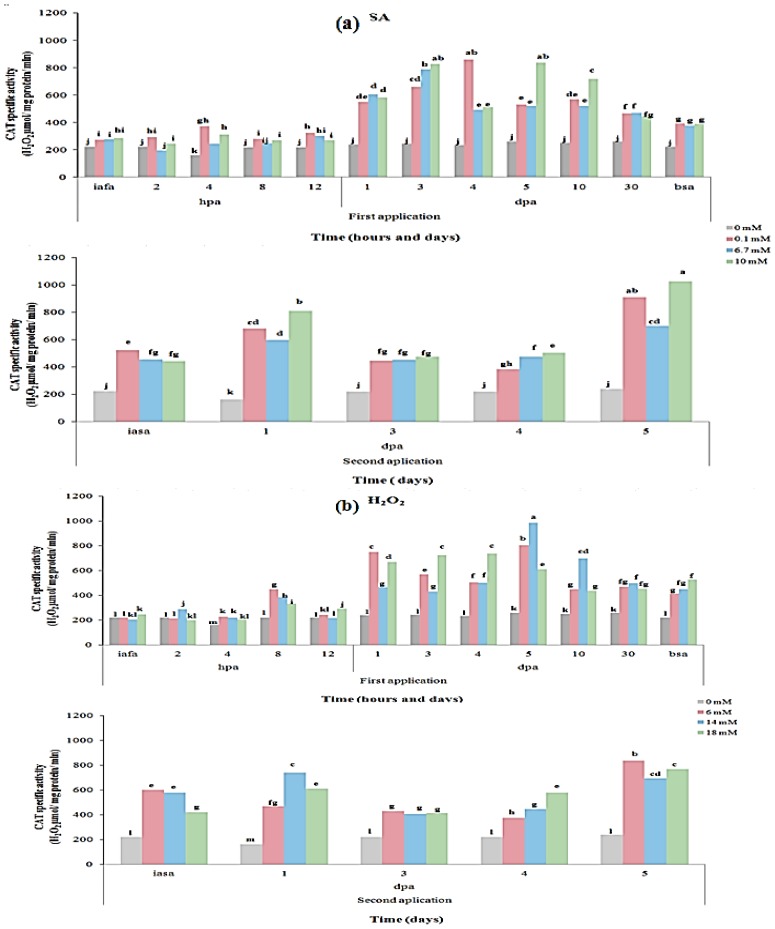

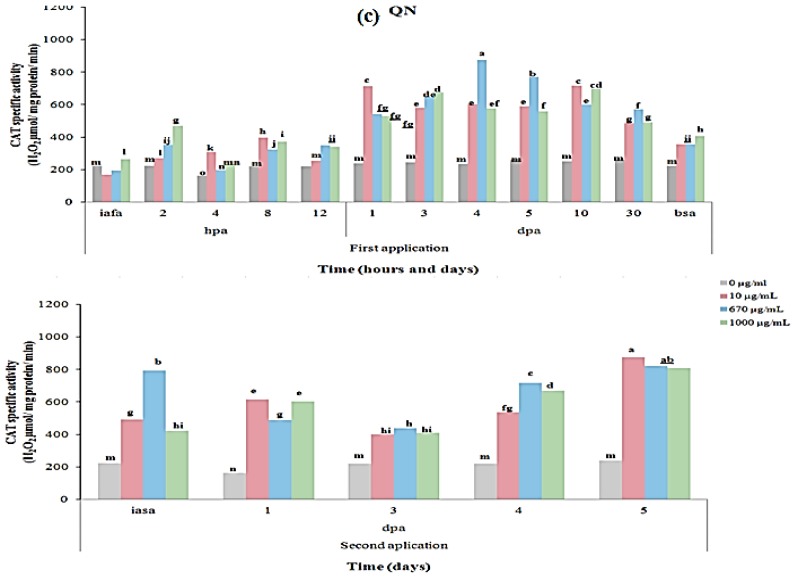
Dynamic from catalase (CAT) specific activity in *C. annuum* L. var. Don Benito as result of unique applications of elicitors at two different times. (**a**) SA elicitor; (**b**) H_2_O_2_ elicitor; (**c**) QN elicitor. Elicitors’ application first in day zero, and second application in day 42 after first application of elicitors. Abreviations: iafa: immediately after first application; bsa: before second application; iasa: immediately after second application; hpa: hours post application; dpa: days post application.

**Figure 4:**
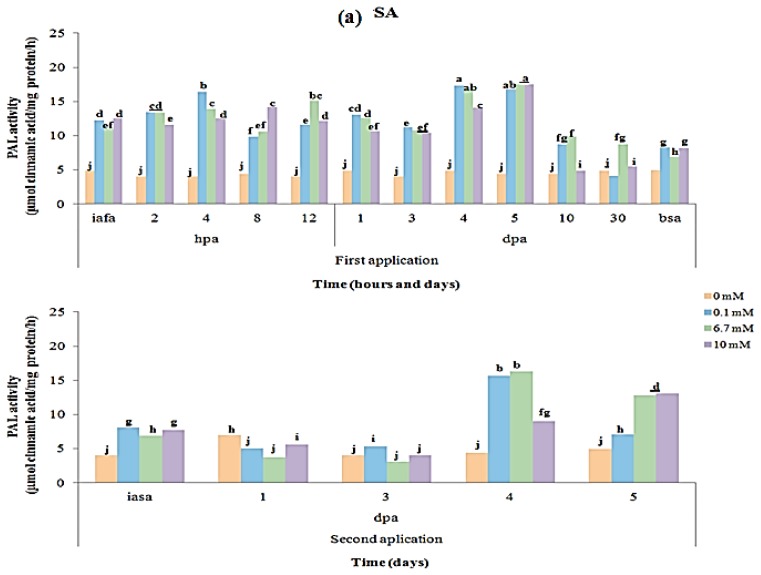

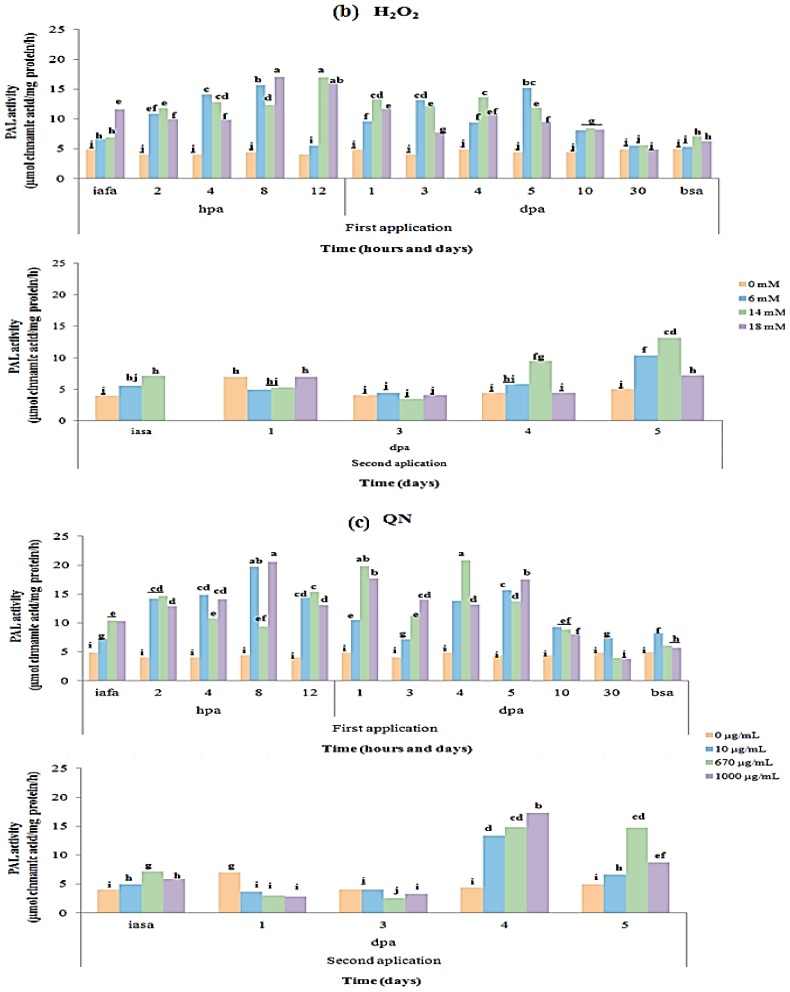
Dynamic from phenylalanine ammonia lyase (PAL) specific activity in *C. annuum* L. variety Don Benito as result of unique applications of elicitors in two different times. PAL specific activity produced by three concentrations of: (**a**) SA elicitor; (**b**) H_2_O_2_ elicitor; (**c**) QN elicitor. Elicitors’ application first at day zero, and second application at day 42 after first application of elicitors. Abreviations: iafa: immediately after first application; bsa: before second application; iasa: immediately after second application; hpa: hours post application; dpa: days post application.

The dynamics of CAT and PAL specific activity in *C. annuum* L. var. Don Benito by elicitors’ application is shown in Figures 3 and 4, respectively. In Figure 3, it can be observed that CAT activity was significantly activated after any of the evaluated elicitors. Additionally, CAT activity followed a similar trend as H_2_O_2_ production level, except during the first 12 h post-application in the first application (Figure 2). In control treatments, CAT activity did not show significant differences. After the second application of elicitors, CAT activity increased at highest levels at 5 dpa (Figure 3). The latter results suggest that CAT activity was directly proportional to the H_2_O_2_ production generated by each elicitor, except during the first hours post-application. Our results suggest that high catalase activity is due to the stimulus received at the plant by elicitors. This coincides with exogenous applications of jasmonic acid (JA, another elicitor) as reported by Liu *et al*. [27], where CAT activity is increased up to 180 U in wheat plants pretreated with 1 mM JA and subsequently subjected to stress with UV-B radiation. Also, Iseri *et al*. [24] demonstrated that exogenous application of H_2_O_2_ in plants of tomato significantly enhance oxidative stress response and tolerance by elevating the antioxidant status of tomato as evidenced by CAT activity. However, our results also differ to those of Airaki *et al*. [28] because they report 100 times less CAT activity than reported in this study, when plants of *C. annuum* are only exposed to cold stress. This difference could be due to which cold stress causes a regular decrease in all the apoplastic antioxidant enzymes (SOD, CAT, and POX) as reported by Mutlu *et al*. [29] in the cold-sensitive cultivar of barley, whereas the SA application to sensitive and tolerant cultivars of barley before the exposure to cold stress increased the activities of the apoplastic antioxidant enzymes.

**Figure 5:**
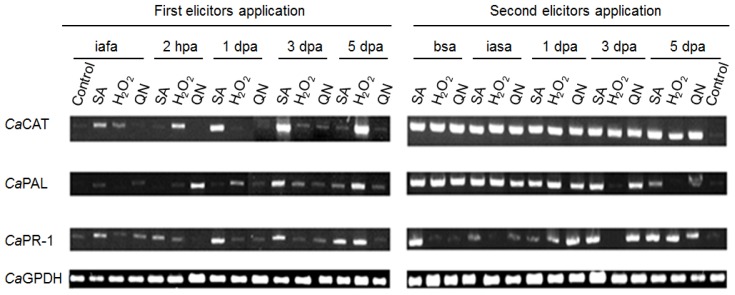
Gene expression in *C. annuum* L. var. Don Benito by effect elicitor’s application. *Cat1*, *pal* and *pr1* expression of *C. annuum* L.var. Don Benito as result of unique applications of SA (10 Mm), H_2_O_2_ (18 mM), and QN (1000 μg/mL) elicitors at two different times. First application at day zero, and second application, at day 42 after first application. Abbreviations: iafa: immediately after first application; bsa: before second application; iasa: immediately after second application; dpa: days post application. Glyceraldehyde Phosphate Dehydrogenase (*Ca*GPDH) gene from *C. annuum* L. was used as control.

On the other hand, PAL is the key enzyme of phenylpropanoid metabolism in higher plants and several studies indicated that the activation of PAL and subsequent increase in phenolic content in plants is a general response associated with disease resistance [30]. PAL activity dynamic by effect of elicitors’ application in *C. annuum* L. var. Don Benito is shown in Figure 4. On the whole, no significant differences in the various sampling times evaluated in control treatments (0 mM elicitor) were shown. In addition, during the first application of any of the evaluated elicitors, significant increases in PAL activity were observed, as displayed in the Figure. This figure shows that PAL activity increases significantly just after application of SA, H_2_O_2_ and QN elicitors, up to 5 days after application, correlating with a H_2_O_2_ production (Figure 2), likely suggesting that this molecule acted as signal for activation of this enzyme as expected. QN increased PAL activity to 21 μg cinnamic acid/mg protein in 8 h (Figure 4c), staying almost unchanged until 4 dpa the first application. SA (Figure 4a) and H_2_O_2_ (Figure 5b) only generated a PAL activity of 17 μg cinnamic acid/mg protein in 4 dpa and 8 hpa, respectively. The results showed that PAL activity was high at 12 h post-application of elicitors (Figure 4), and the fact that this occurs in the first hours post-application suggests the rapid signaling from hydrogen peroxide for activation of this enzyme, which catalyzes the first step in phenylpropanoid biosynthetic pathway, having an important role in several aspects of plant growth, development and in the inducible plant defenses against both biotic and abiotic stresses [31–33]. Finally, after second application of elicitors, a significant increase in PAL activity was displayed after 4–5 days depending on the elicitor and concentration evaluated.

### 2.4. Gene Expression Pattern of *cat1*, *pal*, and *pr1* in *C. annuum L. var.* Don Benito

H_2_O_2_ is involved in the regulation of several stress-related genes, and genes encoding to *cat1* and *pal*, which are widely used as indicators of ROS responsive and oxidative stress specific signaling. On the other hand, *pr1* is indicative of biotic stress [32,33]. The gene expression pattern of *cat1*, *pal*, and *pr1*, by effect of elicitors’ application at different times in *C. annuum* L. var. Don Benito is shown in Figure 5. All expression patterns were evaluated using the highest concentration of each elicitor: SA (10 mM), H_2_O_2_ (18 mM) and QN (1000 μg/mL). It is shown that *cat1* expression is mainly induced by SA (10 Mm) and H_2_O_2_ (18 mM) in the first application of these elicitors. Moreover, Figure 5 shows that *cat1* expression was 20-fold increased respect to control, in the second application of elicitors. The *pal* expression pattern showed that, in the first application of elicitors, SA (10 mM) induced twice as much *pal* expression as the control. QN (1000 μg/mL) induced 4 times as much *pal* expression in both evaluated times. After second elicitor application, the *PAL* expression increased 12-fold with respect to the control, until 3 dpa for SA (10 mM) and QN (1000 μg/mL), the expression of which decreased for day 5 dpa, in contrast with H_2_O_2_ elicitor, which did not maintain a significant *pal* expression in 3 and 5 dpa, with respect to SA and QN. Finally, gene expression of *pr1* was mainly induced with SA (10 mM), with initial expression of 3 times more than the control in the first application and extends during the 5 dpa of the same application, increasing its expression until 12 times that of the control in the second application. Induction of expression with H_2_O_2_ (18 mM) maintained low levels, between 1–2 fold more than the control, in the first application of elicitors. In the second application of elicitors, it can be observed that expression level changed from 1 to 6-fold in the second application. QN induced expression of *pr1* on average 1.3-fold in the early 2 hpa of first application, increasing on average 9.3-fold more than the control in the second application. It should be noted that the *cat1*, *pal* and *pr1* expression from control plants that were sprayed only with water, was at the same level in at all times tested, so we decided to show only a representative image from the control.

The expression of three catalase genes from *Nicotiana plumbaginifolia* has been analyzed, demonstrating that *cat1* is specifically involved in the scavenging of photorespiratory H_2_O_2_, which, with the exception of senescing petals, is restricted to green organs [34]. In addition, based on the analysis of *cat1* expression, transcript level in *C. annum* leaves were increased through oxidative stress dynamic, mainly by SA and H_2_O_2_, and potentially induced with second application (Figure 5). This result indicates that the enhanced catalase activity in systemic leaves of *C. annuum* L. elicitor treated is likely at least in part attributable to the increase in *cat1* expression levels and scavenging of the H_2_O_2_ generated by elicitors’ treatment. The aforementioned is because it is not expected that cat1 is the only gene encoding catalase activities in pepper, as suggested elsewhere [35]. These latter authors showed an increase in *cat1* mRNA level in *C. annuum* L. in paraquat-treated plants. It suggests that *cat1* can play an important role in response to environmental stresses. Regarding *pal* expression, it is known that an increase in the content of *pal* mRNA often underlies activation of the enzyme [36]. As well as the *cat1* expression, the *pal* expression was induced mainly by SA and H_2_O_2_ elicitors, and substantially increased in second elicitor application, which is attributed to the state of alert in which the plant is already in (Figure 5). PR proteins are locally induced in response to pathogen attack as well as systemically in both compatible and incompatible host/pathogen interactions [37]. For instance, the SAR response of pepper plants is accompanied by a systemic microoxidative burst that generates H_2_O_2_ and a systemic expression of defense-related genes in uninoculated leaves [37]. ROS induces the coordinate expression of a set of so-called SAR genes [38]. In this context, De Román *et al.* [39] showed that induced resistance to foliar pathogens with analogous of SA, acibenzolar-S-methyl (ASM) can (**i**) move from the above-ground to the below-ground compartment and (**ii**) affect mutualistic micro-organisms as well as plant pathogens. Our results show the rapid induction of *pr1* of *C. annuum* L. with SA (10 mM) and H_2_O_2_ (18 mM) in contrast with QN (1000 μg·mL^−1^) induction, where *pr1* expression is minimal (Figure 5). Indicating that exogenous application of elicitors is an efficient manner of inducing defense-related genes and proteins. In *Arabidopsis* leaves from plants treated with chitosan, benzothiadiazole, wounding, methyl viologen or control were used for the purification of phosphorylated proteins [40]. These authors described the quantitative changes of phosphoproteins present in *Arabidopsis thaliana* leaves after challenging with elicitors or treatments mimicking biotic stresses, which stimulate basal resistance responses, or oxidative stress.

## 3. Experimental Section

### 3.1. Plant Growth

The variety Don Benito of *C. annuum* L. was utilized in this study. This species was used because in our laboratory, pepper has been a study model for several physiological, molecular and biochemical studies since 10 years ago. The seeds were treated with 200 ppm of potassium nitrate to stimulate germination. After germination, the seedlings were grown in a substrate of peat moss in a germination room with a photoperiod of 16 h light-8 h dark at 25 °C. Plants were watered with the Steiner nutritive solution at 50% [41].

### 3.2. Stress Treatments with Elicitors’ Application

Treatments with elicitors were applied to plants of 8–10 leaf-stage, and the control plants were treated only with water. Two foliar applications unique, were took out at a time deferred with the objective of generate a dynamic of an oxidative stress. Acid salicylic (SA), hydrogen peroxide (H_2_O_2_), and chitosan (QN) were the elicitors used. The reason for using these elicitors was because in our laboratory these elicitors have been studied in order to further agronomic applications in greenhouse plant production. The QN was derived from chitin shrimp, had a molecular weight of 1836.277 g/mol and deacetylation degree of 95% [42]. Three different concentrations of each elicitor were used: 0.1 mM, 6.7 mM, and 10 mM for SA (Sigma, St. Louis, MO, USA); 6 mM, 14 mM, and 18 mM for H_2_O_2_ (Mallinckrodt Reagent Chemicals, Raleigh, NC, USA); and, 10 μg/mL, 670 μg/mL, and 1000 μg/mL for QN [42]. These concentrations were established because they are within the concentrations range where these elicitors have been tested with antimicrobial activity [12,14,43]. The QN solutions were previously hydrolyzed as described by Lizárraga-Paulín *et al.* [44]. Samples were taken immediately after the first application (iafa) of elicitors in day zero, as well as at 2, 4, 8, and 12 h post application (hpa), and at 1, 2, 3, 4, 5, 10 and 30 days post application (dpa). Subsequently, at day 42 after the first application when plants had 12–16 leaf-stage, a second application was made. The sampling was done before and after of this application, as well as at 1, 3, 4 and 5 dpa. The sampling was performed of apical leaves in both phenologial stages of the plants analyzed.

### 3.3. H_2_O_2_ Detection with DAB Staining

The detection H_2_O_2_ in leaves of *C. annuum* L. by using 3,3-diaminobenzidine (DAB) as substrate, is a qualitative method to measure the H_2_O_2_ generation, and was realized according to method described by Thordal-Christenssen *et al*. [18] with the following modifications: plant leaves were cut with a scalpel from the base of the stem, and submerged for 8 h under light at 25 °C in 1 mg/mL solution of DAB-MOPS 10 mM (Sigma-Aldrich, St. Louis, MO, USA), because MOPS is one of the main buffers of biological systems. The solution was acidified with HCl until obtain a pH 3.8 necessary to solubilize DAB. [18] After that time, the sheets were washed with methanol in a warm water bath, and then were stored in 50% glycerol [20,21]. Dark-brown zones indicated the presence of H_2_O_2_.

### 3.4. Plant Extracts Preparation

Plant extracts were obtained in accordance with Sibanda and Okoh [45] with following modifications: 50 mg plant tissue were frozen and milled in liquid nitrogen. Samples were homogenized with two washes in acetone (2.5 mL per wash) and centrifuged at 5000 rpm for 10 min, the supernatant was removed and 1.5 mL of 0.05 M potassium phosphate (pH 7.0) was added to the pellet, which was resuspended through vortex and centrifuged at 13000 rpm for 15 min.

### 3.5. H_2_O_2_ Content Assay

For quantitative measurements of H_2_O_2_ production, 100 μL of plant extract was mixed with Hydrogen Peroxide Substrate Solution (90 μL) containing ferrous iron and xylenol orange (Hydrogen Peroxide Assay Kit, National Diagnostics Atlanta, GA, USA), the blank was prepared in the same manner except that 100 μL of 0.05 M potassium phosphate (pH 7.0) was used instead of the sample. The mixture was incubated at room temperature for 30 min. The absorbance at 560 nm was measured for each sample and compared with a standard curve made by measuring known hydrogen peroxide concentrations. Experiments were performed in duplicate.

### 3.6. Enzyme Activity Assays

CAT activity was determined spectrophotometrically, monitoring the oxidation of H_2_O_2_ at 240 nm, as described by Chandlee *et al.* [46]. The reaction mix consisted of 1 mL of plant extract and 1 mL of 0.022 M H_2_O_2_. The blank was prepared in the same manner except that 1 mL of 0.05 M potassium phosphate (pH 7.0) was used instead of the sample. An aliquot of the extract was used to determine protein content through the Bradford method [47] utilizing bovine serum albumin as standard. The enzyme-specific activity is expressed as μmol of oxidized H_2_O_2_ per mg of protein, per minute (μmol/mg protein/min).

To determine PAL activity, plant extracts were prepared similarly using 0.1 M borate buffer (pH 8.8). PAL activity was determined spectrophotometrically at 290 nm by the formation of trans-cinnamic acid (SIGMA) as the method described by Gerasimova *et al.* [48] with some modifications. The standard curve was performed using different concentrations of cinnamic acid. The reaction mix contained 100 μL of plant extract and 100 μL 60 μM/mL l-phenylalanine (MERCK, Naucalpan, Edo. Mexico, Mexico) solution. Reaction mixes were incubated at 37 °C for 1 h. In control samples, the extract was replaced by borate buffer. The reaction was stopped by adding 50 μL 1 M trichloroacetic acid (J.T. Baker, Phillipsburg, NJ, USA). Protein concentration was measured according to the method described by Bradford [47]. Enzyme activity was expressed by the amount of cinnamic acid produced in μmol/mg protein/h.

### 3.7. Gene Expression Analysis of *cat1*, *pal*, and *pr1* in *Capsicum annuum* L

Gene expression analysis was carried out at 0, 24, 72, and 120 h, and at day 42 after first elicitor’s application. Also took place at 0, 24, 72, and 120 h after the second elicitor application, carried out in day 42 after first elicitors application, with objective know if it maintains or increases the gene expression after the second application of elicitors and their relationship with the H_2_O_2_ production dynamics by effect of elicitors in *C. annuum* L. Total RNA extraction was done using TRIzol^®^ Reagent (Catalogue 15596-026, Invitrogen, Carlsbad, CA, USA). Complementary DNA (cDNA) was obtained through First Strand cDNA Synthesis Kit (Catalogue K1611, Fermentas, Glen Burnie, MD, USA), starting from 700 ng of total RNA. To carry out amplification of genes *cat1*, *pal* and *pr1* of *Capsicum annuum* L., were obtained its sequences of the GenBank database to design specific oligonucleotides (Table 1). Also, glyceraldehyde phosphate dehydrogenase gene (GPDH) from *C. annuum* L. (accession number AJ246011) was evaluated. PCR conditions were: 30 cycles of 95 °C for 30 s; 65 °C for 2 min, and 72 °C for 1 min. PCR products were visualized on 1.5% agarose gels using a digital image system (DNR Bio-Imaging Systems Mini BIS Pro, Hamisha, Jerusalem, Israel).

### 3.8. Statistical Analyses

Statistical analysis was conducted using the software JMP 5.0.1 [49]. A completely random experimental design was used to evaluate the effect of the type and concentration of elicitors in the two varieties of *C. annuum* L. for oxidative stress studies. The arrangement consisted of 20 treatments with 3 replications, considering as experimental unit 4 plants. Data were subjected to analysis of variance (ANOVA) and the differences between means were compared using Tukey’s test (*p* < 0.05).

## 4. Conclusions

Exogenous application of SA, H_2_O_2_, and QN elicitors in *C. annuum* L significantly increased endogenous H_2_O_2_ as well as gene expression and enzymatic activities related with plant defense as phenylalanine ammonia lyase and catalase 1. The duration of oxidative and molecular inductions was 30 days in the first elicitors’ application. The second elicitors’ applications displayed significant increased activity of CAT and PAL and H_2_O_2_ endogenous concentration after 4–5 days post-application in an elicitor-dose manner. It can be suggested that monitoring the biochemical and molecular indicators evaluated in this work might be a criteria to determine the appropriate time for elicitors’ application, and thus induce the defense system of *C. annuum* L. for agricultural uses. Based on our results, elicitor application could be conducted once a month to keep on alert the plant defense system, in order to diminish the cost of an induced response that is typically measured as a reduction in plant fitness. In our laboratory, currently we are undertaking experiments related to plant-costs for elicitation, especially using metabolomic and physiological methods. Also, we will attempt to evaluate all these aspects related to elicitors’ application in agricultural practices, with minor plant and environmental-costs and increased quality of microbe-free products.

## Figures and Tables

**Table 1:** Sequences of oligonucleotides used to evaluate the molecular response of the application of elicitors in *C. annuum* L.

Name	Oligonucleotide sequence (5′-3′)	Product size (bp)	Genbank accession No.
***Ca*CAT-F**	GTCCATGAGCGTGGAAGCCCCGAAT	841	AF227952
***Ca*CAT-R**	CGCGATGCATGAAGTTCATGGCACC		
***Ca*PAL-F**	TGGTGGATTTTTCGAGTTGCAGCCG	831	EU616575
***Ca*PAL-R**	TGGCAAAGCGCCACGAGATAGGTTG		
***Ca*PR1-F**	CTTTTGCTATATTTCACTCAACACAAGCCC	522	AF053343
***Ca*PR1-R**	TGCTGGATTTATTTTCCTTTTAACACATGA		
***Ca*GPDH-F**	GGCCTTATGACTACAGTTCACTCC	255	AJ246011
***Ca*GPDH-R**	GATCAACCACAGAGACATCCACAG		
